# SLE presenting as migratory arthritis, chorea and nephritis

**DOI:** 10.31138/mjr.29.1.43

**Published:** 2018-03-19

**Authors:** Sajal Ajmani, Durga Prasanna Misra, Able Lawrence

**Affiliations:** Sanjay Gandhi Postgraduate Institute of Medical Sciences, Lucknow, Uttar Pradesh, India

**Keywords:** Pediatric SLE, lupus, chorea, migratory arthritis, anti-phospholipid antibodies

## Abstract

We present a case of a 12-year-old girl who presented with migratory arthritis, chorea and ascites. She was diagnosed to have systemic lupus erythematosus (SLE) and subsequently responded to immunosuppressive therapy. She had been misdiagnosed earlier as having rheumatic fever. Our case highlights the fact that SLE should be considered in the differential diagnosis of a patient with migratory arthritis & chorea. Generally, chorea in SLE is immune-mediated rather than due to ischemia and has good prognosis.

## INTRODUCTION

Movement disorders such as parkinsonism, dystonia, tics and tremor are rare in systemic lupus erythematosus (SLE).^1^ Of these, chorea is the most commonly reported, with a frequency between 1% and 4%.^2^ Chorea in SLE has been found to be associated with antiphospholipid (aPL) antibodies. The serologic profile of aPL antibodies varies; most reports indicate that immunoglobulin G (IgG) anticardiolipin (aCL) is the main antibody related to lupus chorea,^2^ while others have emphasized the importance of lupus anticoagulant in these patients.^3^ This case highlights the need to consider SLE as a rare but important differential diagnosis in patients presenting with chorea with past history of migratory arthritis.

## CASE PRESENTATION

A 12-year-old girl presented with a history of pain and swelling in multiple joints for one year and fever associated with involuntary movements for 4 months. She was apparently healthy until one year ago, when she developed migratory arthritis involving the left knee joint at first followed by the right knee, right elbow and left shoulder. The pain and swelling lasted for 7–10 days before involving the other joints. She had been diagnosed to have acute rheumatic fever and started on analgesics and penicillin prophylaxis. The joint symptoms subsided in three months. For the past 4 months, she had history of fever (documented to be around 101–102° F) occurring every 3^rd^ day with chills and rigor. Along with fever, she had developed involuntary movements of all 4 limbs (more on the left side). The involuntary movements tended to disappear during sleep. The abnormal movements on the right side had decreased while they persisted on the left. She also gave a history of pedal edema and periorbital swelling for the past 4 months. For the last week prior to the assessment, she had abdominal distention, passing of frothy urine and multiple and painless oral ulcers. There was no history of skin rash, Raynaud’s phenomenon, hematuria, altered sensorium, cough, shortness of breath, palpitations, syncope or jaundice. Her mother had died in her early twenties due to a similar illness with symptoms of involuntary movements, swelling of the feet and fever.

On examination, she was pale with a pulse rate of 100/min (regular, all pulses palpable) with blood pressure of 160/90 mm Hg in the right upper limb. She had bilateral cervical and axillary lymphadenopathy. They were firm, mobile, with the largest lymph node measuring 2 x 2 cm. She had facial swelling and symmetrical, pitting pedal edema. There was choreoathetosis (more on the left than the right) and hypotonia in all limbs, bilateral flexor plantar response and normal deep tendon reflexes. Abdomen was distended with presence of shifting dullness. Rest of the general physical and systemic examination were normal.

**Figure 1.**
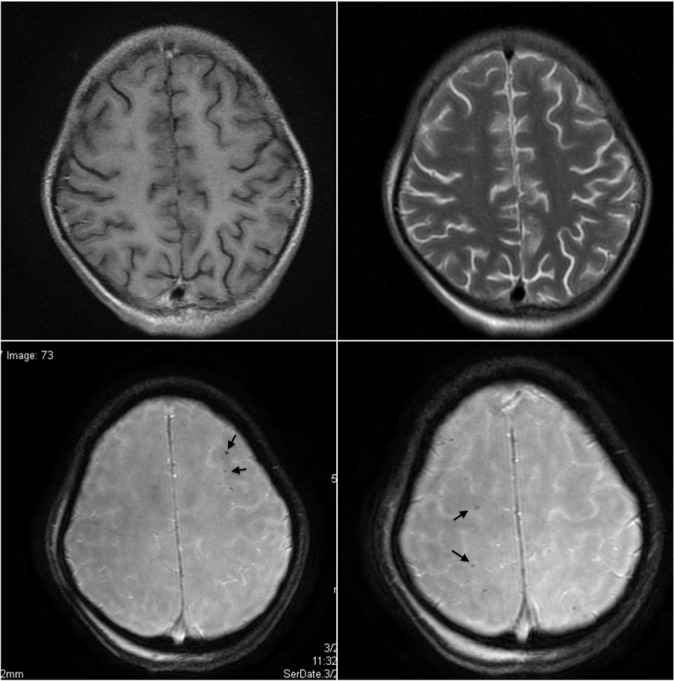
MRI BRAIN with T1 T2 (top left and right) and gradient echo (GRE, below) sequences. GRE sequences showing multiple foci of tiny punctate hemorrhage (Blooming on GRE; marked with arrow) in right fronto-parietal and left frontal regions.

Investigations revealed anemia (hemoglobin 5.9g/dl), total leucocyte count 8000/mm^3^, differential leucocyte count – neutrophils 81%, lymphocytes 12% (absolute lymphocyte count-960/mm^3^), platelets- 209,000/mm^3^, reticulocyte count- 3%, peripheral blood smear showed normocytic normochromic anemia, serum bilirubin-1.2mg/dl, aspartate aminotransferase 18 U/L, alanine aminotransferase 16 U/L, serum alkaline phosphatase 76 U/L, serum protein- 5.1 g/dl with albumin- 2.3 g/dl, serum creatinine- 1.7 mg/dl, blood urea- 130 mg/dl. Urine examination showed 4+ protein, 50–60 red cells per high power field (HPF), 40–50 white cells/HPF, erythrocyte sedimentation rate 123 mm/hr, serum C-reactive protein 1.90mg/dl. Antinuclear antibody (ANA) by immunofluorescence was 4+ homogenous (1:80 dilution), anti-double stranded deoxyribonucleic acid (dsDNA) antibodies were elevated (>300 IU/ml), serum complements were low (C3- 39.3 mg/dl [60–120], C4 9.5 mg/dl [15–25]). Lupus anticoagulant was positive, however, anti- β2 glycoprotein, IgG & IgM anti-cardiolipin antibodies were negative. Coomb’s test (direct and indirect) were negative. Workup for other causes of chorea at this age were non-contributory [serum ASO titer negative (<200IU), echocardiogram normal, serum ceruloplasmin- 25.4mg/dl (20–35), 24-hour urinary copper-28μg (20–50)]. Her MRI brain (**Figure 1**) showed microbleeds suggestive of small vessel involvement; however, prothrombin time and activated partial thromboplastin time were within normal limits.

She had oliguria at admission (urine output 300 ml/day) with an elevated serum creatinine of 2.1mg/dl. A diagnosis of lupus nephritis with chorea and secondary anti-phospholipid antibody syndrome was made. She was administered 5 daily pulses of methylprednisolone (15/mg/kg), two sessions of hemodialysis and started on monthly cyclophosphamide as per the National Institutes of Health (NIH) protocol(4) for lupus nephritis. Subsequently, her urine output improved to 1600ml/day and serum creatinine normalized to 1 mg/dl by the sixth day of admission. Her chorea also decreased within one week and disappeared completely within one month.

## DISCUSSION

Our patient presented with fever, choreoathetosis, ascites, decreased urine output and a history of migratory arthritis. Differential diagnosis considered were SLE, rheumatic fever and Wilson’s disease. However, only lupus could explain all the abnormalities. The diagnosis was confirmed on the basis of lymphopenia, active urinary sediment, positive ANA, elevated anti-dsDNA antibodies and hypocomplementemia.

In children with rheumatic fever, joint symptoms usually subside within one month of onset, later followed by chorea (1–6 months after the streptococcal infection). However, in lupus, the joint pain and fever can be concurrent with the onset of chorea. The presence of classical features such as malar rash, oral ulcers, hair loss, nephritis, present in our case, also suggested the diagnosis of SLE rather than rheumatic fever or Wilson’s disease. We could identify another published report of a young female with lupus who presented with chorea and arthritis and had been misdiagnosed as having rheumatic fever.^5^ Athetosis, whenever present, is generally seen in combination with chorea in patients with SLE.^7^ We could not find reports of isolated athetosis without chorea.^7^

Two possible mechanisms (ischemic or immune-mediated dysfunction in basal ganglia) possibly drive the pathogenesis of chorea in SLE with aPL antibodies. However, this is predominantly driven by immune mechanisms.^6^ In a previously published series of thirty-two patients of SLE with chorea, twelve (37.5%) had normal brain MRI, whereas only three patients had unilateral T2 lesions of the basal ganglia in the caudate nucleus.^7^ Studies in animal models have shown that aPL can result in depolarization of synaptoneurosomes.^8^ Furthermore, contralateral striatal hypermetabolism has been previously reported in a patient with primary APS and alternating hemichorea.^9^ These studies lend credence to the hypotheses that direct activation of the basal ganglia circuits could possibly be mediated by IgG aPL antibodies causing ischemia with interruption of the cortical-basal ganglia-thalamus-cortical circuit, or it could be the result of vascular damage driven by long-term SLE with associated vascular risk factors.

Different therapeutic strategies have been described in literature to treat chorea in lupus. In the absence of any other indication for immunosuppression, observation or treatment with dopamine antagonists alone may be successful and chorea has been known to resolve with time.^10^ Patients who do not respond and are markedly symptomatic should be treated with immunosuppressants, anti-coagulation or both of these depending upon the imaging and the clinical scenario. Our patient had severe nephritis along with chorea, hence, we treated her with intravenous monthly cyclophosphamide following NIH protocol for lupus nephritis.

In conclusion, lupus can rarely present with a movement disorder with choreoathetosis and antiphospholipid antibodies are often found in such patients. It is also important to remember that SLE can present with migratory arthritis. Our patient had a good outcome with immunosuppression.
